# Ward and Clinical Work

**Published:** 1910-09-03

**Authors:** 


### WARD AND CLINICAL WORK.

Having successfully negotiated his qualifying examination in anatomy and physiology, the student must at once decide whether he intends proceeding to some higher examination in these subjects, *e.g.* the primary examination for the diploma of Fellow of the Royal College of Surgeons of England, or whether he intends devoting his attention to the medical side of his profession. If the former be his object, there can be no doubt that it is best for him to proceed at once to the further study of anatomy and physiology which such an examination entails. There is no advantage in, and many disadvantages accrue from, putting off the examination until after qualification. The additional learning he may have picked up in the wards will be of little use to him in examinations in anatomy and physiology, and he will find that his knowledge of these subjects has sadly deteriorated during the two years he has spent in clinical work. It is also usually a mistake to enter for examinations in pathology and bacteriology before doing a certain amount of work in the wards. Even if such tests be successfully surmounted, the student will find that his knowledge of these subjects is not at its best at a time when he stands most in need of it. There can be no doubt that examinations passed in this way tend to make the student leave the subjects on one side, and forget them, whereas an intelligent knowledge of them is of the greatest importance in the final examinations in medicine and surgery. These subjects should, therefore, be studied along with the ordinary text-books, and not crammed up for an examination and then forgotten.

Assuming, however, that the student either has passed his higher examination in anatomy and physiology, or has decided to dispense with it, his attention is next turned to the wards and clinical work. Most hospitals, before allowing him to enter the wards, insist on his attending a course of lectures on clinical methods. At these he is taught the elementary working procedures of medicine and surgery, the way to apply bandages, test urines, examine patients, etc. These are very necessary preliminaries to attendance in the wards, and should be followed assiduously in order to prevent waste of time, mistakes, and disagreements subsequently. The house officers under whom the student will work are busy men, whose time is precious, and who naturally resent having to repair deficiencies in the student's education occurring through his own fault. The position of the student who, when told, say, to apply a fomentation to Number 6's leg, or to test the urine of Number 9 for sugar, has to confess that he does not know how to do it, is not enviable. All this can be avoided by an intelligent attendance at these preliminary lectures. It is well, before commencing ward work, to purchase a book containing information on clinical methods, and to read it carefully. Such a book has been written by Drs. Hutchison and Rainy, is called "Clinical Methods," and is published by Cassell and Co., Ltd., price 10s. 6d. It will be found of the greatest use, especially in the medical wards. Having obtained a sufficient knowledge of general principles, the student enters the wards. He is appointed either dresser to a surgeon, or clerk to a physician of the hospital. There is no apparent reason why one of these appointments should be taken before the other, but a custom has grown up whereby the new student is as a rule given surgical work to do first. The appointment is usually given for three months, and it is perhaps best that the student, on completing this period, should forsake surgical for medical work. The first three months spent in the study of each subject are of necessity not so profitable as the second. It is advisable that the less profitable periods of each subject should be got through in succession, so that a general foundation of knowledge may be laid in each. Greater advantage can then be taken of opportunities which occur in the second periods.

Again it is well to realise that no such sharp line of demarcation exists between medicine and surgery as might on the face of things seem to be the case. Surgical diseases are met with in medical beds, and *vice versâ*, at times. It is only the degree of the affection or questions of convenience in nursing which determine to which side of the hospital patients are sent. A proper grounding in both arts should therefore be obtained before completing the necessary six months' ward work in either. It should be borne in mind that a good house-officer can be of the greatest assistance to the keen student. Therefore in many cases it is a good practice, in choosing the physician or surgeon under whom to work, to take note of the particular house-officer he may have under him at the time, rather than be led to a decision by the eminence of his chief in the medical world. The house-officer, like the poor, is always with you, whereas his chief is only to be seen at fitful intervals—the more fitful, perhaps, the greater his eminence. It is not always the eminent who are the best teachers, and in any case the knowledge that a man has been considered fit to be on the staff of a teaching hospital should be guarantee enough that the student will profit by his teaching. Moreover, it will be possible, when the necessary appointments have been held, to listen to the words of wisdom of the eminent one at the clinical lectures and demonstrations given for those students about to take their final examinations.

While attached as dresser or clerk to some member of the staff, the student will have to attend the courses of lectures in surgery and medicine prescribed by the rules of the hospital. Attendance at these is required before a certificate is granted entitling him to sit for his final examination. The question now arises as to what should be the student's next move after fulfilling his first appointments in medicine and surgery. This is not easy to answer, but, on the whole, if he be going in for the examinations of the Conjoint Board, he had perhaps best turn his attention to midwifery and the diseases peculiar to women. The elementary knowledge of medicine and surgery he has gained in his first six months' work will stand him in good stead when acting more or less on his own on "the district." He will, therefore, attend the necessary lectures entitling him, on their completion, to be appointed midwifery clerk to the hospital. Having looked after the essential twenty or more confinement cases, he will, if possible, get an appointment as clerk in the gynæcological wards for three months, during which time he will, in addition to his work therein, attend the out-patient department if possible, and revise his knowledge of midwifery. He should then, by the end of his first year, be in a position to take his examination in midwifery and gynæcology, the first part of his final. If, however, he be a university candidate, he will be obliged to delay this until he is in a position to enter for his examination in surgery, for, as a rule, midwifery and gynæcology form part of the surgical examinations at universities. The student has now come to his second year of study. In this he must complete his appointments in medicine and surgery, attend a fever hospital for two or, in the case of university students, for three months, act as post-mortem clerk for one month, attend a course of lectures on vaccination and a similar course on the diagnosis and treatment of mental disorders, as well as put in lectures and practical work in pathology and bacteriology, if he has not already done the latter.

In addition, he will be expected to take more than a casual interest in the practice of the special departments connected with diseases of the eye, ear, nose, throat, skin, etc. There is no reason why he should take the appointments in any particular order, but it is perhaps as well to attend the fever hospital before putting in a second period of three months as clerk in the medical wards, for opportunities arise at times of seeing fever cases, such as measles and typhoid, in these wards. In any case, attendance at the fever hospital can be coupled with advantage with an appointment as post-mortem clerk. It is, however, as can easily be seen, not advisable to attend the fever hospital while taking part in work which necessitates the frequenting of the wards or out-patient department. It is true that every precaution is taken by the authorities at the fever hospitals for the prevention of the spread of infection by third persons; but it is better to take no risks, and in any case most surgeons would not allow a fever-student to cross the threshold of their wards. The time thus thrown on one's hands by this enforced abstinence from clinical work can, however, easily be filled up by increased energy in the acquirement of knowledge of pathology and bacteriology. Practical work in the laboratory can readily be made to fit in with attendance at the fever hospital, and if any time is still left over it can be filled up in the post-mortem room and the museum. When the student has fulfilled all the obligations imposed upon him by the regulations governing the particular qualification he is desirous of acquiring, he is at liberty to sit for the final examination in medicine and surgery. He may prefer to take this in parts or as a whole. No guidance can be offered in such a matter, as it depends on the capacity of the individual student. At most hospitals papers are set by members of the teaching staff in all the subjects of the final examination, and until a student has successfully negotiated these tests he will not be given leave to present himself for the qualifying examination.

Much has already been said as to work in the wards and medical school, but nothing so far of play. The student who is eager to qualify quickly and well will find that play will have to be restricted to the minimum compatible with health. Long holidays are things of the past for such a man. A fortnight at Christmas, a month in the summer, a week at Easter, and an occasional week-end are the absolute maximum he can allow himself in the year of regular holidays. He will usually be able to get Saturday afternoons and Sundays off, but his duties may even necessitate his sacrificing some of these at times. In winter golf is a very good game to play, as it does not entail the extreme exhaustion caused by playing football and hockey in a half-trained condition. In summer tennis and, if procurable, boating hold the palm, though, of course, the keen golfer will deny this last assertion. Cricket has its charm for some, but regular practice is difficult to obtain, partly owing to the difficulties in transit, at any rate as regards London, and partly owing to the fact that students holding an appointment often cannot get away from hospital sufficiently early to make it worth while going to the nets. Swimming is an excellent exercise, and good baths are plentiful in most towns. Those of more sedentary habits will find chess and rifle clubs attached to most schools. On the whole, however, play is necessarily subordinate to work, and only the really clever man can hope to qualify in the minimum time if he indulges his passion for sport to any great extent.

